# Regulation of soluble epoxide hydrolase in renal-associated diseases: insights from potential mechanisms to clinical researches

**DOI:** 10.3389/fendo.2024.1304547

**Published:** 2024-02-15

**Authors:** Peng Gao, Yongtong Cao, Liang Ma

**Affiliations:** Department of Clinical Laboratory, China-Japan Friendship Hospital, Beijing, China

**Keywords:** soluble epoxide hydrolase, sEH inhibitors, renal diseases, epoxyeicosatrienoic acids, sEH polymorphisms

## Abstract

In recent years, numerous experimental studies have underscored the pivotal role of soluble epoxide hydrolase (sEH) in renal diseases, demonstrating the reno-protective effects of sEH inhibitors. The nexus between sEH and renal-associated diseases has garnered escalating attention. This review endeavors to elucidate the potential molecular mechanisms of sEH in renal diseases and emphasize the critical role of sEH inhibitors as a prospective treatment modality. Initially, we expound upon the correlation between sEH and Epoxyeicosatrienoic acids (EETs) and also addressing the impact of sEH on other epoxy fatty acids, delineate prevalent EPHX2 single nucleotide polymorphisms (SNPs) associated with renal diseases, and delve into sEH-mediated potential mechanisms, encompassing oxidative stress, inflammation, ER stress, and autophagy. Subsequently, we delineate clinical research pertaining to sEH inhibition or co-inhibition of sEH with other inhibitors for the regulation of renal-associated diseases, covering conditions such as acute kidney injury, chronic kidney diseases, diabetic nephropathy, and hypertension-induced renal injury. Our objective is to validate the potential role of sEH inhibitors in the treatment of renal injuries. We contend that a comprehensive comprehension of the salient attributes of sEH, coupled with insights from clinical experiments, provides invaluable guidance for clinicians and presents promising therapeutic avenues for patients suffering from renal diseases.

## 1 Introduction

The interplay between soluble epoxide hydrolase (sEH) and epoxyeicosatrienoic acids (EETs) plays a crucial role in regulating various physiological processes. Arachidonic acid (AA), a polyunsaturated fatty acid, is metabolized by cytochrome P450 enzymes to form EETs, which serve as endogenous signaling molecules with diverse activities including vascular regulation, anti-inflammation, antioxidation, and tissue regeneration (1–3). sEH, encoded by the EPHX2 gene, modulates EET bioavailability by converting them into diol metabolites (4). Inhibiting sEH, either through genetic deletion or pharmacological intervention, leads to elevated levels of EETs, resulting in a spectrum of protective effects (5), so sEH has emerged as a potential pharmacological target for kidney diseases. Moreover, sEH exerts regulatory effects on renal diseases through mechanisms involving oxidative stress, inflammation, endoplasmic reticulum (ER) stress, and autophagy (6–8). This intricate interplay between sEH, EETs, and their impact on renal physiology and pathology forms a critical nexus in understanding and potentially intervening in various renal-associated diseases.

In this review, we first clearly introduce the relationship between sEH and Epoxyeicosatrienoic acids (EETs), enumerate common typical EPHX2 single nucleotide polymorphisms (SNPs) associated with renal diseases and investigate the sEH-regulated potential mechanisms. Subsequently, we introduce the clinical research on inhibition of sEH for regulation of renal-associated diseases, including acute kidney injury, chronic kidney diseases, diabetic nephropathy and hypertension-induced renal injury. Our goal is to prove the possible role of sEH inhibitors in the treatment of renal injury. In our opinion, a detailed understanding of the key characteristics of sEH and its clinical experiment provides useful information for clinicians and offers potential therapeutic options for renal disease patients.

## 2 sEH and EETs

Arachidonic acid (AA) is a 20-carbon polyunsaturated fatty acid, which is typically esterified in membrane phospholipids to form the second carbon. Phospholipase A2 specifically recognizes the sn-2 acyl bond of phospholipids and catalyzes the hydrolysis of membrane phospholipids to release AA. Cytochrome P450 (CYP) enzymes are used to metabolize AA to EETs in renal tubular cells. Many CYP enzymes can perform the epoxidation of AA, and CYP2C and 2J are related to the formation of kidney EETs, with 11,12-EET being the predominant epoxide (9). As an endogenous polarizing factor, EETs have a wide range of physiological activities, including regulating vascular tension, glomerular hemodynamics, anti-inflammation, antioxidation, anti-platelet aggregation, promotion of sodium excretion, and organ and tissue regeneration (10).

The human sEH is encoded by the gene EPHX2, which is located to chromosomal region 8p21-p12 (11), which consisted of 19 exons encoding 555 amino acids (12). sEH is a member of the epoxide hydrolase (EH) family, which is a bifunctional enzyme with lipid epoxide hydrolase and lipid phosphatase activities. It consists of an N-terminal phosphatase and a C-terminal hydrolase separated by a short proline-rich linker (13, 14). The C-terminal domain hydrolyzes epoxides to their corresponding diols by adding water to the three-membered oxirane ring (15). The N-terminal domain has phosphatase activity for hydrolyzing lipid phosphates (14) and shows specificity for fatty acid diol phosphates (16). The confirmed substrates of the sEH phosphatase domain *in vitro* also include sphingosine-1-phosphate (SIP), lysophosphatidic acid (LPA), and various poly-isopentenyl phosphates including farnesyl pyrophosphate, geranylgeranyl pyrophosphate, and farnesyl monophosphate (17). sEH is widely expressed in many tissues throughout the body, especially in the liver, kidney, intestine, and vasculature (18–20). Additionally, sEH is found in human brain tissue cells such as neurons, astrocytes, oligodendrocytes, and ependymal cells (9). In human kidney tissue, sEH is highly expressed in the renal cortex (21) and more concentrated in the renal microvasculature and proximal tubule (18, 22).

**Figure 1:**
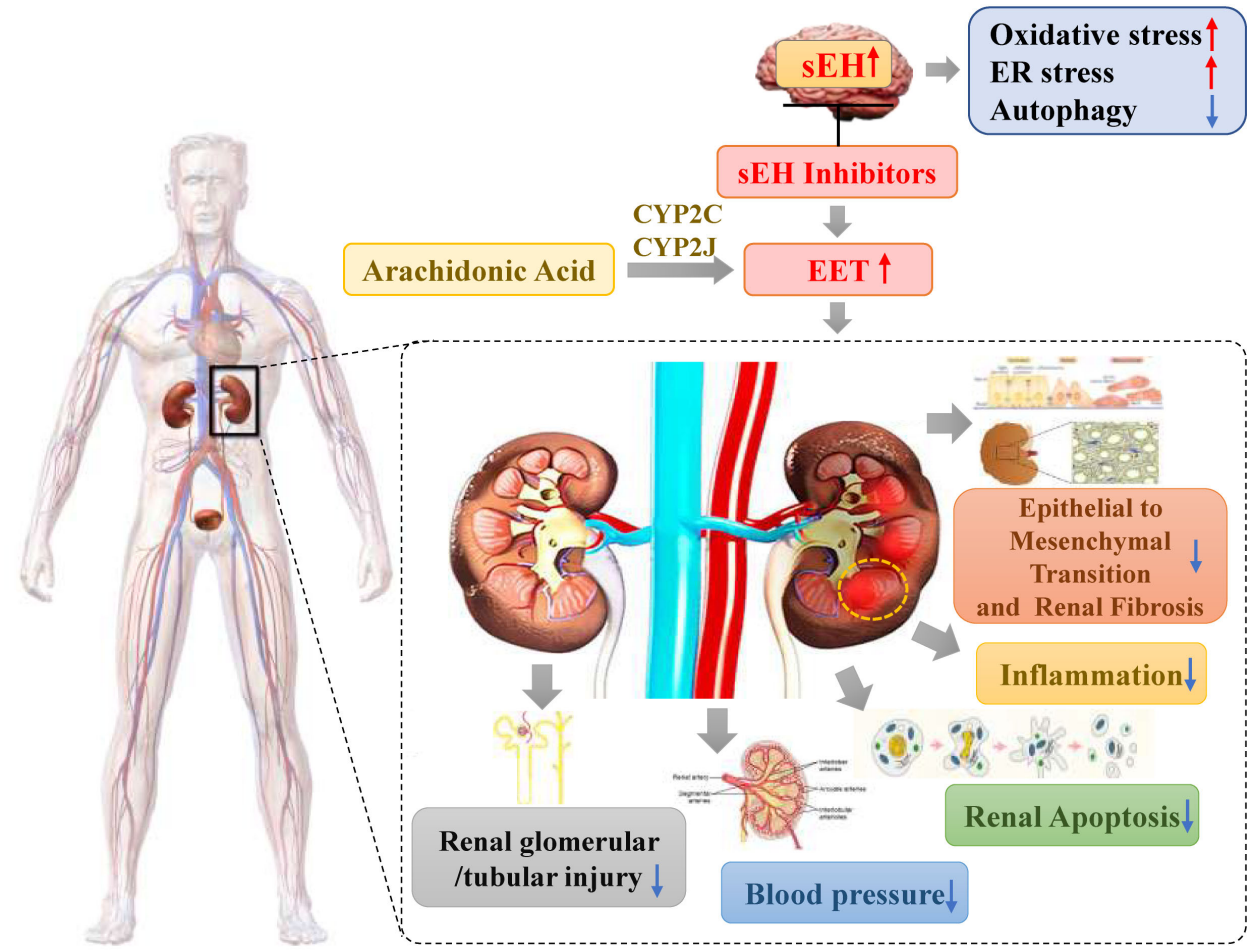
The reno-protection effect of sEH inhibition.

The activity of sEH is thought to be the main determinant of EET bioavailability (10, 18, 23). Conversion of EET to dihydroxyeicosatrienoic acid (DHETs) by sEH is the main pathway of EET metabolism (15). This attenuates most functional effects of EETs, making sEH a logical target for increasing and prolonging the actions of EETs. sEH inhibition decreases DHET formation and leads to intracellular EET accumulation. This results in more EET incorporation into phospholipids and utilization by other metabolic pathways, including b-oxidation and chain-elongation. Functional responses are increased because of the larger amounts of intracellular unesterified EET and EET-containing phospholipids. Furthermore, more EET is released when intracellular phospholipids are hydrolyzed, maintaining the increased intracellular concentration of unesterified EET (15). Numerous researches have shown that either the genetic deletion or pharmacological inhibition of sEH can decrease blood pressure (24), suppress inflammatory response (5), attenuate histological damage, and alleviate the progression of renal tubulointerstitial fibrosis in diabetic nephropathy, hypertensive nephropathy, and unilateral ureteral obstruction models (6, 22, 23). Due to its potential role in kidney diseases, sEH is being pursued as a potential pharmacological target. The reno-protection effect of sEH inhibition is shown in the **Figure 1**.

Some studies suggest sexual dimorphism in EETs levels (25–28). Sexually dimorphic of sEH was firstly identified in a study which sEH activity was found to be remarkably higher in tissues of male and ovariectomized female mice compared to intact females (27, 29). Another study showed that knockout of the Ephx2 gene (sEH-KO) or treatment with sEHIs in male mice reduced their blood pressure to the level comparable to that of wild-type (WT) females. In the latter, disruption of the Ephx2 gene further reduced blood pressure but with significantly smaller decrement than in male counterparts (28, 30). Studies have elucidated, through *in vivo* and *in vitro* models, that estrogen silences sEH transcriptional activity by methylation of the Ephx2 gene promoter. This process involves multiple regulatory signals driven by transcription factors, providing the basic mechanism explanations for the sexually dimorphic expression of sEH (26). Female-specific adaptations were observed in male sEH-KO mice, suggesting that estrogen downregulation of sEH duplicates the actions of Ephx2 deletion, resulting in identical patterns of attenuated coronary myogenic responses, enhanced coronary perfusion and improved cardiac contractility. This is consistent with similar cardiac EET metabolic profiles among female WT, male sEH-KO mice and male WT mice treated with sEHIs (30, 31). Studies using animal models of cerebral ischemia demonstrated that estrogen suppression of sEH was responsible for the female-favorable protection against cerebral ischemic damages in an EET-dependent manner (25). Collectively, the increase in EETs provides better cardiovascular performance and a lower incidence of ischemic diseases in women.

Furthermore, the expression of sEH and the levels of EETs also impact macrophage infiltration and polarization. Current research indicates that the CYP2J2-EETs-sEH metabolic pathway maintain metabolic and immune homeostasis by regulating the polarization of adipose tissue macrophages, ultimately alleviating inflammation and associated insulin resistance, which is related to the inhibition of the cAMP-EPAC signaling pathway (32). *In vitro* study showed that EETs inhibited the polarization of lipopolysaccharide (LPS)-induced M1 macrophage polarization, and reduced pro-inflammatory cytokines production. Simultaneously, EETs preserved the expression of M2 macrophage markers and increased anti-inflammatory cytokine IL-10. EETs also downregulated the activation of NF-κB and upregulated peroxisome proliferator-activated receptors (PPAR α/γ) and heme oxygenase-1 (HO-1). In a mouse model of LPS-induced cardiac dysfunction, recombinant adeno-associated virus (rAAV)-mediated CYP2J2 expression increased EETs levels, alleviating LPS-induced cardiac dysfunction and inflammation. Therefore, CYP2J2/EETs regulates macrophage polarization and becomes potential therapeutic applications in inflammatory diseases (33). Another study explored the relationship between the expression of sEH and macrophage polarization in IgA nephropathy. The study found a positive correlation between sEH expression levels, proteinuria, and macrophage infiltration in the kidneys. Upregulation of sEH promoted M1 polarization of macrophages, while inhibition of sEH and supplementation with EETs reversed this effect, promoting M2 polarization. Thus, inhibiting sEH could be a potential strategy to prevent inflammation and alleviate renal tubulointerstitial fibrosis (34).

## 3 sEH and other epoxy fatty acids

Eicosapentaenoic acid (EPA) and Docosahexaenoic (DHA) have been shown to be highly efficient alternative substrates of CYP epoxygenases, leading to the formation of epoxyeicosatetraenoic acids (EEQs) and epoxydocosapentaenoic acids (EDPs or EpDPEs), respectively (35, 36). CYP epoxygenases selectively catalyze the epoxidation of the terminal double bond of ω-3 PUFAs, leading to the predominate formation of 17,18-EEQ from EPA and 19,20-EDP from DHA (37). In cells, EDPs and EEQs are rapidly metabolized by cytosolic sEH enzyme that similarly metabolizes other epoxy fatty acids including the EETs, to form their corresponding vicinal diol dihydroxyeicosapentaenoic acids (diHEPEs) and dihydroxyeicosatetraenoic acids (diHETEs), respectively (38). Since the products are as a rule generally far less active than their epoxide precursors, so these epoxides operate as short-lived signaling agents that regulate the function of their parent or nearby cells.

Compared with EETs, EEQs and EDPs have similar or more potent effects for vasodilation, anti-inflammation and analgesia (39–44). 17,18-EEQ, as well as 14–15-EET, inhibited TNF-α-induced inflammation in human bronchi via NF-κB- and PPAR-γ-related mechanisms (40, 41). In a rat model of carrageenan-induced inflammatory pain, it was observed that all epoxygenated polyunsaturated fatty acids (EETs, EEQs, and EDPs) demonstrated inhibitory effects on inflammatory pain. However, the potency of the anti-inflammatory effects of EEQs was found to be comparatively lower than that of EETs and EDPs (44). In terms of vasodilation, EDPs are among the most potent vasodilators ever discovered (42). Research of murine hypertension model of angiotensin-II dependent hypertension suggest that EPA and DHA epoxy metabolites contribute to the reduction of systolic blood pressure and alleviation of inflammation by decreasing prostaglandins and MCP-1. Additionally, they contribute to lowering blood pressure and mitigating inflammation by upregulating the expression of ACE-2 in angiotensin II-dependent hypertension (43).

Furthermore, these epoxy fatty acids and their metabolites also play a certain positive role in kidney diseases. 19,20-EDP shows potential therapeutic effects in reducing renal fibrosis. In a unilateral ureteral obstruction (UUO) mouse model, treatment with 19,20-EDP resulted in a 40-50% reduction in renal fibrosis, decreased collagen-positive areas and hydroxyproline content, and reduced renal fibrosis by inhibiting epithelial-mesenchymal transition (EMT) (45). In a chronic kidney disease (CKD) rat model, providing a diet rich in ARA and DHA slowed down urinary albumin excretion and reduced levels of plasma lipid peroxides (LPO), indicating that the combination of ARA and DHA could inhibit the progression of early-stage CKD (46). The significant increase in EPA, EEQs, and the dihydroxy metabolites of these epoxides in serum and urine induced by these diets may be a contributing factor to the improvement of renal diseases.

## 4 sEH polymorphisms associated with renal diseases

Some sEH single nucleotide polymorphisms (SNPs) that lead to amino acid substitutions have been identified in human populations (47–49), including Lys55Arg, Arg103Cys, Cys154Tyr in the sEH lipid phosphatase region and Arg287Gln, Val422Ala and Glu470Gly in the sEH Lipid epoxide hydrolase domain (49). A study has demonstrated that EPHX2 Arg287Gln and the double mutant Arg287Gln/Arg103Cys showed significantly reduced epoxide hydrolase activity, resulting in an sEH enzyme with 25–58% and 11-18% inadequate catalytic activity, respectively. While the Lys55Arg and the Cys154Tyr mutants tended to have increased epoxide hydrolase activity (49). Furthermore, when compared to the most frequent allele, Arg287Gln, Arg103Cys, Lys55Arg and the Cys154Tyr variants have significantly reduced phosphatase activity. This indicates that these polymorphisms influence both the N-terminus and the C-terminus domains, and these domains are not entirely independent of each other (49, 50). A study on the relationship between EPHX2 Arg287Gln and DN in Chinese patients with type 2 diabetes mellitus showed that there was a significant correlation between Arg287Gln and homocysteine (Hcy) levels and DN risk. The A allele of EPHX2 rs751141 exon polymorphism was negatively correlated with the incidence rate of DN, which may be regulated by homocysteine levels (51). The research that explored the relationship between EPHX2 functional variants and acute kidney injury (AKI) after cardiac surgery showed that the Arg287Gln variant was not associated with AKI, while EPHX2 Lys55Arg was associated with AKI after cardiac surgery in patients without previous chronic kidney disease (CKD) (52). The detailed information of EPHX2 polymorphisms is shown in the **Table 1**.

**Table 1:** The detailed information of some common human EPHX2 gene polymorphisms.

Mutant	dbSNP	Alleles	Chromosome (GRCh37)	Exon in EPHX2 gene	Frequency in ExAC	Epoxide hydrolase activity compared to MAF	Phosphatase activity compared to MAF	Enzyme stability
Lys55Arg	rs41507953	A>G	8:27358505	Exon 2	G=0.09008/10913	Increase	Decrease	Decrease
Arg103Cys	rs17057255	C>T	8:27361241	Exon 3	T=0.014243/1715	No statistically significant	Decrease	Decrease
Cys154Tyr	rs57699806	G>A	8:27362587	Exon 5	A=0.003695/447	Increase	Decrease	Decrease
Arg287Gln	rs751141	G>A	8:27373865	Exon 8	A=0.115323/13999	Decrease	Decrease	Decrease
Val422Ala	rs531961160	T>C	8:27396198	Exon 14	C=0.000008/1	No statistically significant	No statistically significant	Decrease
Glu470Gly	rs68053459	A>G	8:27399019	Exon 16	G=0.00112/136	No statistically significant	No statistically significant	Decrease
Arg287Gln/Arg103Cys	rs751141/rs17057255	G>A/ C>T	8:27373865/ 8:27361241	Exon 3/ Exon 8	A=0.115323/13999/ T=0.014243/1715	Decrease	Decrease	Decrease

## 5 Potential mechanisms regulated by sEH in renal diseases

Research indicates that modulating sEH content, like through EET, mitigates inflammation and safeguards kidney function. Reduced sEH content correlates with lower ER stress, decreased autophagy, and reduced NF-κB-induced inflammation. Autophagy, enhanced with sEH deficiency, plays a protective role in kidney function. The following will explore the potential regulatory mechanisms of sEH in kidney disease from three aspects: oxidative stress and inflammation, ER stress, and autophagy.

### 5.1 Oxidative stress and inflammation

Previous studies and experiments have confirmed that oxidative stress can effectively activate NF-κB, which promotes the expression of inflammatory genes in the body of diabetic patients (53). In an experiment involving gene upregulation expression, researchers tested the effects of overexpressed CYP2J2, CYP2C8 and sEH on the genomes of knockout mice. Experimental results show that EET has a certain anti-inflammatory effect, can effectively inhibit the functional activation of NF-κB, and reduce the protein expression level of cytokines (7). Therefore, based on this result, oxidative stress symptoms and cellular inflammation can be improved by controlling sEH content, effectively reducing the degradation of EET and better protecting the normal operation of kidney function.

### 5.2 ER stress

Studies have proven that the lack of sEH content in the body is due to a decrease in the ER stress response and an increase in autophagy behavior, resulting in a decrease in the degree of NF-κB-stimulated inflammatory factor production and the development of fibrosis. Lack of sEH content and high glucose-induced podocyte autophagy are caused by decreased ER stress, a process consistent with the effect of sEH content in regulating the degree of ER stress (54, 55). Decreased podocyte autophagy and ER stress attenuate the activation of hyperglycemic-induced NF-κB inflammatory factor. In addition, there is a link between ER stress response and the pathogenesis of DN (56), and relief of ER stress response and associated molecular chaperones can appropriately alleviate the severity of diabetic nephropathy (57, 58). Therefore, a reasonable definition of podocyte sEH deficiency can start from the renal protective function of patients with hyperglycemia, mediated by reduced ER stress.

### 5.3 Autophagy

Autophagy occurs in cells as a protective mechanism. Macromolecular components are degraded or recycled by phagocytizing one’s own protein cells or organelles. Autophagy prevents cell damage and responds to toxic stimuli of cells, which is a self-protective mechanism for cells (59). It has been shown that the enhancement of autophagy behavior in podocytes is related to the decrease in sEH content, and several elements (Beclin, LC3-I/II and Atg5/7) are labeled with protein labeling techniques. It can be found that these labeled elements do play a significant role in the development and maturation evolution of autophagosomes. This finding is the same as the increased autophagy behavior that has been reported due to symptoms of sEH deficiency (8, 60). With the continuous demonstration of experimental phenomena, autophagic cells play a great role in regulating kidney function and ensuring kidney function. Some studies have shown that the STZ-induced diabetic disease model can also effectively mimic the diabetes-inducing chemicals in laboratory animals (61, 62). Enhanced autophagy responses have been observed in diabetic mice and in epithelial cells cultured with nutrients with higher glucose content (63, 64). It is undeniable that the lack of sEH in podocytes in the body can cause cells to enhance the autophagy response, thereby exhibiting certain protective and stressful behaviors against podocyte damage.

## 6 Inhibition of sEH or co-inhibition of sEH with other inhibitors for the regulation of renal-associated diseases

Effectively inhibiting the level of sEH or co-inhibition of sEH with other inhibitors can have a good protective effect on renal-associated diseases, such as AKI, CKD and DN, which has been effectively verified in several researches.

### 6.1 Inhibition of sEH as a single target for the regulation of renal-associated diseases

The complex pathophysiological processes of AKI include hemodynamic changes, inflammation, endothelial dysfunction, and damage to tubular epithelial cells (65–67). Many studies have investigated their role in AKI through genetic disruption of the Ephx2 gene or chemical inhibition of sEH. In a C57BL/6 mouse model of renal ischemia-reperfusion injury (IR), it has been confirmed that targeting sEH may reduce the risk of AKI. This was achieved by controlling sEH activity through intraperitoneal injection of the sEH inhibitor AUDA. Administration of a sEH inhibitor prior to IR attenuated renal functional decline, tubular necrosis, and renal inflammation and the severity of IR- induced renal damage correlated inversely with endogenous EET levels (68). The current study established protective effects of podocyte-specific sEH deficiency against LPS-induced renal injury. Bettaieeb et al. have reported in a lipopolysaccharide (LPS)-induced mouse model, and they believe that mRNA protein expression levels in mouse podocytes increase significantly when LPS attacks. Podocyte sEH-deficient mice experienced less renal impairment than controls with normal renal function (69). Podocyte-specific sEH disruption notably alleviated LPS-induced kidney dysfunction, and this was associated with reductions in NF-kB inflammatory response, MAPK, and ER stress signals, indicating that sEH inhibition in podocytes may have potential therapeutic implications for combating podocyte injury (69). Additionally, research showed that soluble epoxide hydrolase (sEH) inhibitor, n-butylester of 12-(3-adamantan-1-yl-ureiido)-dodecanoic acid (nbAUDA), can attenuate cisplatin-induced acute nephrotoxicity (70). EET hydrolysis was significantly reduced in Ephx2(-/-) mice and correlated with the attenuation of elevated serum blood urea nitrogen and creatinine levels induced by cisplatin. Histological evidence of tubular injury and neutrophil infiltration in Ephx2(-/-) mice was also reduced. Similarly, cisplatin had no impact on renal function, neutrophil infiltration, or tubular structure and integrity in mice treated with the potent sEH inhibitor AR9273 (71). PTUPB effectively reduces sorafenib-induced glomerular nephrotoxicity. PTUPB can lower blood pressure and proteinuria, alleviate tubular and fibrotic damage, and improve glomerular health (72). These data suggest that inhibiting sEH can alleviate chemotherapeutic agent-induced kidney injury. However, there are also studies indicating that the absence of sEH may have detrimental effects in AKI. In a mouse model of unilateral ischemia-reperfusion injury induced after acute non-renal excision in the remaining kidney, sEH gene disruption did not improve I/R-induced kidney damage but rather exacerbated renal functional impairment, tubular injury, and inflammatory response (73).

Due to the complexity of its pathogenesis, treatment for CKD has always been challenging. Tubulointerstitial fibrosis is the primary pathway in CKD that leads to disease progression and ultimately results in End-Stage Renal Disease (ESRD). Inhibiting sEH is a potential CKD treatment strategy. In a CKD murine model of type 1 diabetes, sEH inhibition improved renal endothelial function and reduced renal injury and inflammation (74). sEH inhibitors have potential applications in the treatment of fibrogenesis in the CKD unilateral ureteral obstruction (UUO) model. sEH inhibitor t-TUCB promoted anti-inflammatory and fibro-protective effects in UUO kidney, prevented tubular damage, downregulated NF-kB, transformed growth Factor-β1/Smad3, and affected inflammatory signaling pathways, while also activating PPAR subtypes. The increased levels of EET due to sEH deficiency also prevented renal interstitial inflammation and fibrosis (75). Research has found that sEH inhibition improves proteinuria-induced renal tubular epithelial-mesenchymal transition (EMT) by regulating the PI3kt-GSK-3b signaling pathway. In the *in vitro* experiments of proteinuria-induced renal tubular EMT, E-cadherin expression decreased, while α-smooth muscle actin (α-sma) expression increased, and its morphology transformed into a myofibroblast like phenotype. In chronic proteinuria nephropathy rat model, the sEH inhibitor AUDA treatment suppressed the activation of PI3K-Akt and phosphorylation of GSK-3b, simultaneously reducing the levels of EMT markers (76). Furthermore, sEH may be a promising preventive target for CKD-associated vascular calcification. *In vivo* and *in vitro* experiments have suggested that the absence of sEH may inhibit vascular calcification (77). However, there are also studies indicating that sEH inhibition may have detrimental effects in CKD. In a CD1 mouse model where ischemic AKI progressing to CKD, treatment with the sEH inhibitor TPPU effectively controlled elevated blood pressure and glomerulosclerosis, but it enhanced renal perfusion injury, leading to increased inflammation and tubulointerstitial fibrosis (78).

Many studies have confirmed that sEH inhibition has a potential therapeutic effect on DN. In streptozotocin-induced DN mouse models, deficiency mice in the sEH gene exhibited reduced diabetes manifestations. The excretion levels of Hb A1c, creatinine, blood urea nitrogen, and urinary microalbumin excretion were significantly decreased. The apoptosis of renal tubules in sEH deficient mice was also reduced, which is consistent with an increase in the levels of Bcl-2 and Bcl-xl that resist apoptosis and a decrease in the levels of Bax that promote apoptosis. These effects are related to the activation of the PI3K Akt NOS3 and AMPK signaling cascades. sEH inhibition and exogenous EETs significantly protected HK-2 cells from TNFα-induced apoptosis (79). In another study on STZ-induced DN mice, sEH inhibitor t-AUCB reduced glomerular albumin permeability. Since albumin and glomerular alpha3 integrin levels can be maintained stably in diabetes rats, the expression of nephron protein is reduced, thus reducing kidney damage. The treatment of t-AUCB has also been shown to protect partial renal function in db/db mice and reduce HK-2 cell apoptosis under high glucose exposure (80). Furthermore, another study has shown that sEH inhibition with t-AUCB can alleviate kidney damage in db/db mice, partially restore autophagic flux, improve mitochondrial function, reduce renal ROS generation, and alleviate endoplasmic reticulum stress. The sEH inhibitor t-AUCB plays a protective role in hyperglycemia induced proximal renal tubular injury, and the potential mechanism of t-AUCB mediated protective autophagy is involved in the regulation of mitochondrial function and endoplasmic reticulum stress (81). Increased expression of sEH protein was observed in the glomeruli of high-fat diet and STZ-induced hyperglycemic mice. Notably, podocyte-specific sEH deficiency preserved kidney function and glucose control, mitigating hyperglycemia-induced renal injury. The beneficial effects of podocyte sEH deficiency were associated with decreased ER stress, enhanced autophagy with a corresponding attenuation in inflammation and fibrosis (82). The beneficial effects of podocyte-specific sEH deficiency suggest that sEH inhibition may have therapeutic significance for hyperglycemia induced renal injury and DN.

Many studies have confirmed the antihypertensive and renal protective effects of sEH inhibitors in angiotensin-dependent hypertension. The renal damage and inflammation caused by salt-sensitive hypertension can be improved by inhibiting the degradation of epoxides, which is related to the hydrolase domain of the Ephx2 gene. Ephx2 gene deficiency can lower blood pressure, alleviate renal inflammation, and improve glomerular damage in patients with DOCA salt-induced hypertension. Therefore, the use of sEH inhibitors provides a dual protection against blood pressure and inflammation, which can alleviate the progression of ESRD associated with salt-sensitive hypertension (83). Previous studies have indicated that the levels of sEH protein in the kidneys of Ang type II hypertensive patients are elevated, which is associated with increased levels of urinary 14,15-DHET in the urine. These studies suggest that the long-term use of selective sEH inhibitor CDU can increase EET levels and lower arterial blood pressure in Type II hypertension animal model. The fact that CDU leads to diuresis, increased urinary EET, and decreased urinary 14,15-DHET excretion rate supports the idea that renal vasodilation and an increase in natriuretic EET may be the potential reasons for sEH inhibition and antihypertensive effect (3). Studies showed long-term sEH inhibition on renal vascular function, vascular, and glomerular damage induced by angiotensin infusion indicates that sEH protein in renal micro vessels is elevated in patients with angiotensin-induced hypertension. Chronic administration of CDU can lower blood pressure and improve renal damage associated with angiotensin-induced hypertension, providing protection for renal vasculature and glomeruli. In the angiotensin-induced hypertension Sprague-Dawley rat model, CDU treatment resulted in reduced urinary albumin excretion (84). In a hypertensive GK rat model, the sEH inhibitor AUDA can inhibit the increased albumin excretion caused by hypertension, prevent morphological changes in the kidneys induced by hypertension, and inhibit the infiltration of monocytes/macrophages into the kidneys, reducing the expression of MCP-1 (85). Another study used sEH inhibitor AUDA to treat angiotensin-sensitive hypertension rats, which led to decreased urinary microalbumin levels and the number of ED-1 positive cells. sEH inhibition can lower blood pressure in patients with angiotensin-sensitive hypertension and improve renal damage (86).

### 6.2 Inhibition of sEH synergizes with other inhibitors for the regulation of renal-associated diseases

#### 6.2.1 Dual inhibitors of sEH and COX

It has been demonstrated that simultaneous augmentation of CYP450-derived EETs along with COX inhibition exerts an additive response attenuating LPS-induced pain and hypotension (87, 88). Besides, CYP450-derived EETs, especially 8,9-EETs, can be further metabolized by COX enzymes to angiogenic 11-hydroxy-8,9-EETs (89, 90). The single molecule sEH/COX-2 dual inhibitor, PTUPB, can lower blood pressure and proteinuria, alleviate tubular and fibrotic damage, and improve glomerular health (72). In type 2 diabetic obese ZSF1 rats, PTUPB reduced renal cytokine expression, decreased immune cell infiltration, and reduced production of chemokine MCP-1 to alleviate kidney inflammation. In *in vitro* studies of isolated renal glomeruli, PTUPB alleviated renal inflammation in DN and also directly affected the glomerular filtration barrier. PTUPB effectively alleviated diabetes-induced kidney injury with DN associated with hyperlipidemia and obesity (91). Additionally, PTUPB resulted in a 30-80% reduction in renal injury parameters and a 25-57% decrease in inflammation and oxidative stress markers in type 2 diabetic rats, indicating PTUPB has a protective effect on metabolic abnormalities and renal function (92).

#### 6.2.2 Dual inhibitors of sEH and PPAR

Several studies highlighted an extensive crosstalk between effects mediated by EETs and peroxisome proliferator-activated receptor (PPAR) signaling (1). PPARs play multiple roles in lipid and glucose homeostasis, however, among these effects, the anti-inflammatory and oxidative stress-reducing properties of EETs which are associated with PPARγ activation, are of special importance (93, 94). RB394 is an equipotent PPARγ-selective full agonist and sEH inhibitor with a favorable pharmacokinetic and pharmacodynamic profile. A study explored the mitigation of renal fibrosis using RB394 in UUO model, the results showed that RB394 alleviated renal fibrosis by reducing kidney inflammation, oxidative stress, tubular injury, and vascular injury (95). Another study was conducted using rat models of the metabolic syndrome and type 2 diabetes. The results showed that RB394 was effective in preventing metabolic syndrome phenotypes, reducing fasting blood glucose and HbA1c levels, improving glucose tolerance, reducing blood pressure, improving lipid profiles, and reducing liver fibrosis and hepatosteatosis. RB394 also demonstrated positive effects in treating diabetic nephropathy by reducing renal interstitial fibrosis and renal tubular and glomerular injury (96). The findings suggest that RB394 is a promising molecule for treating renal-associated diseases.

## 7 Clinical trials on sEH inhibitors

Numerous relevant experimental data from preclinical animal models show that sEH inhibition can effectively improve renal-associated diseases. This is the reason that sEH inhibitors can be widely used in clinical trials. Two well-established sEH inhibitors have been used in human clinical trials (84, 97, 98). The effects of AR9281 and GSK2256294 are evident (99, 100), but AR9281 may not have a sufficiently high therapeutic effect in clinical trials for hypertension and the treatment of type 2 diabetes (99). In addition, there is a sEH inhibitor is GSK2256294A, which works by weakening cell activity and inhibiting the conversion rate of 14,15-EET to 14,15-DHET in human, rat and mouse whole blood. GSK2256294 has entered a human clinical trial to evaluate the treatment of diabetes mellitus and metabolic disorders (ClinicalTrials.gov ID: NCT03486223). EC5026 is an orally active sEH inhibitor to resolve inflammation and neuropathic pain without the addictive potential of opioids (101). Two phase 1a clinical trials of EC5026 (ClinicalTrials.gov ID: NCT04908995 and ClinicalTrials.gov ID: NCT04228302) have demonstrated favorable safety. Another Phase 1b multiple ascending dose (MAD) study is in process (ClinicalTrials.gov ID: NCT06089837) to investigate the safety, tolerability, and pharmacokinetics (PK) of two sequential dose regimens of oral EC5026 in healthy volunteers. The commonly used sEH inhibitors have been shown in the **Table 2**.

**Table 2:** The chemical properties for some commonly used sEH inhibitors.

sEH inhibitors	Activity	Water solubility	Half-life	Model	Clinical research	Reference
TPPU 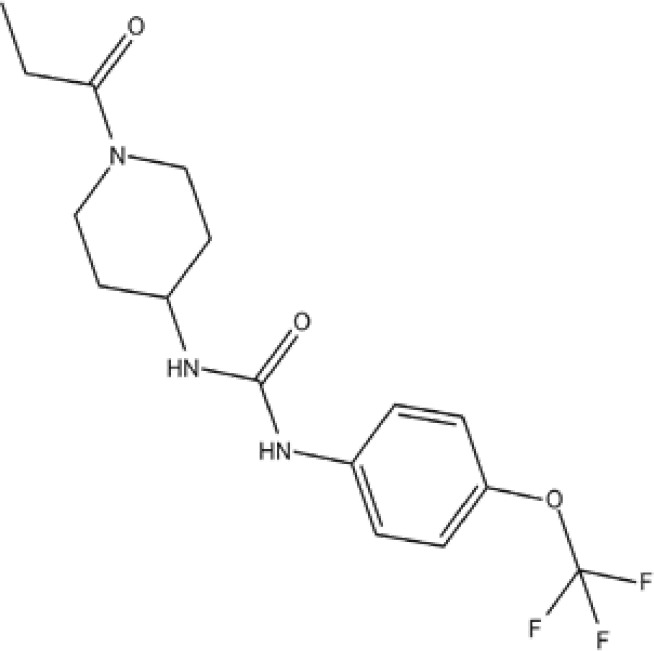	high activity on the primate sEH, good activity with rodent sEH and often poor activity on sEH of other species.	low	long	CD1 mice in a model of renal IRI	effectively controled elevated blood pressure and glomerulosclerosis	(78)
t-AUCB 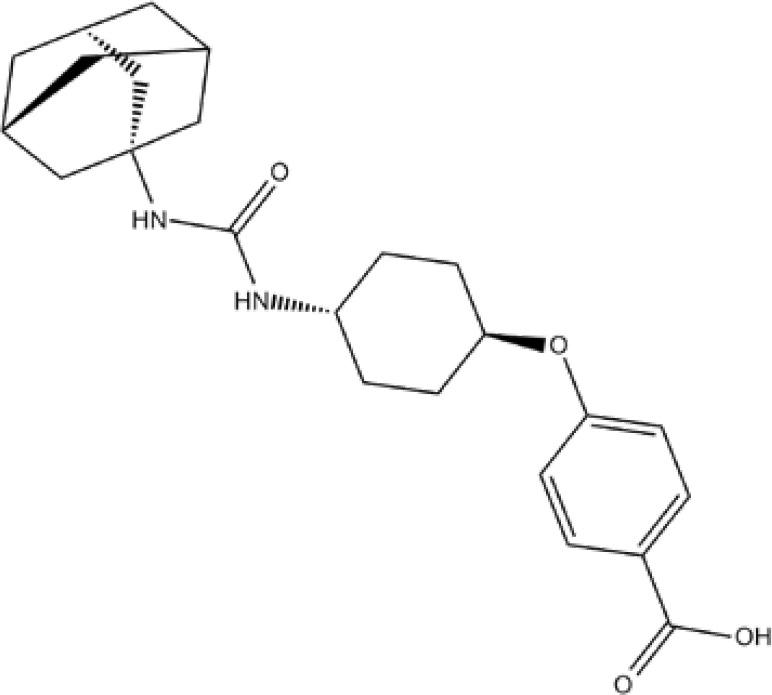	good potency on sEH from a variety of species.	high	short	Streptozocin-induced DN mouse model	reduced glomerular albumin permeability	(80)
t-TUCB 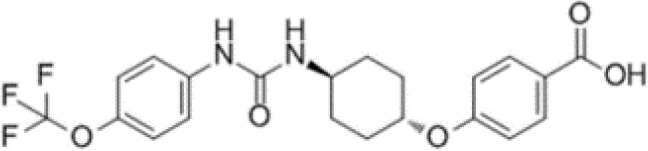	good potency on sEH from a variety of species.	high	long	unilateral ureteral obstruction (UUO) and glomerulonephropathy model	promoted anti-inflammatory and fibro-protective effects in UUO kidney, prevented tubular damage, downregulated NF-kB, transformed growth Factor-β1/Smad3, and affected inflammatory signaling pathways	(75)
RB394 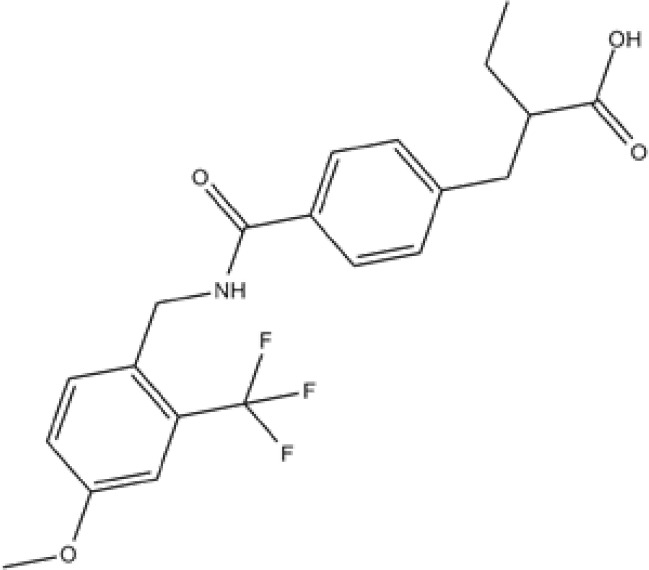	good potency on sEH from a variety of species.	high	long	UUO model	alleviated renal fibrosis by reducing kidney inflammation, oxidative stress, tubular injury, and vascular injury	(95)
AUDA 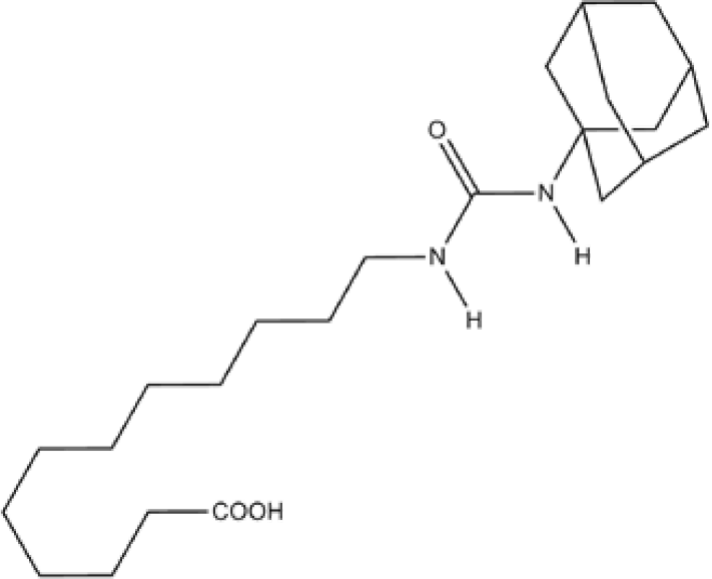	moderate potency on sEH from a wide variety of species.	high	short	murine renal ischemia-reperfusion injury model	attenuated renal functional decline, tubular necrosis, and renal inflammation	(68)

## 8 Conclusion

In this review, we first briefly introduced the sEH, including its structure, distribution, substrates, and physiological functions. We listed some typical EPHX2 single nucleotide polymorphisms (SNPs) and elaborated on their potential effects on sEH. Next, we outlined the potential mechanisms regulated by sEH from three aspects: oxidative stress and inflammation, ER stress, and autophagy. Subsequently, we introduced the *in vivo* and *in vitro* experiments involving sEH inhibition associated with various types of renal injury, as well as recent clinical trials of sEH inhibitors. Our aim is to determine the potential role of sEH inhibitors in the treatment of renal diseases.

Numerous preclinical animal models have provided evidence of the efficacy of sEH inhibition in renal injury, considering sEH as a prominent therapeutic target. Besides, clinical trials of sEH inhibitors for other diseases have not yielded exciting results, and some of the clinical trials have proved to be ineffective. It is worth noting that not all studies have shown the beneficial effect of sEH inhibition on kidney diseases. Jung et al. reported that sEH inhibition with t-AUCB failed to elicit protective effects in the 5/6 nephrectomy mouse model and notably aggravated proteinuria (102). Thus, the role of sEH in diverse kidney diseases needs to be further elucidated by future studies, and many more mechanistic studies are required to enable extrapolation of animal results to clinical applications.

## Author contributions

PG: Validation, Writing – review & editing, Conceptualization, Resources, Writing – original draft. YC: Validation, Writing – review & editing, Formal analysis, Supervision. LM: Formal analysis, Validation, Writing – review & editing, Data curation, Funding acquisition.
